# OrchidBase 4.0: a database for orchid genomics and molecular biology

**DOI:** 10.1186/s12870-021-03140-0

**Published:** 2021-08-12

**Authors:** Yu-Yun Hsiao, Chih-Hsiung Fu, Sau-Yee Ho, Chung-I Li, You-Yi Chen, Wan-Lin Wu, Jeen-Shing Wang, Di-Yang Zhang, Wen-Qi Hu, Xia Yu, Wei-Hong Sun, Zhuang Zhou, Ke-Wei Liu, Laiqiang Huang, Si-Ren Lan, Hong-Hwa Chen, Wei-Sheng Wu, Zhong-Jian Liu, Wen-Chieh Tsai

**Affiliations:** 1 Orchid Research and Development Center, National Cheng Kung University, Tainan, 70101 Taiwan; 2 Department of Electrical Engineering, National Cheng Kung University, Tainan, 70101 Taiwan; 3 Department of Statistics, National Cheng Kung University, Tainan, 70101 Taiwan; 4 Department of Life Sciences, National Cheng Kung University, Tainan, 70101 Taiwan; 5 Institute of Tropical Plant Sciences and Microbiology, National Cheng Kung University, Tainan, 70101 Taiwan; 6 Key Lab of National Forestry and Grassland Administration for Orchid Conservation and Utilization at College of Landscape Architecture, Fujian Agriculture and Forestry University, Fuzhou, 350002 Fujian China; 7 Zhejiang Institute of Subtropical Crops, Zhejiang Academy of Agricultural Sciences, Wenzhou, 325005 China; 8 School of Life Sciences, Tsinghua University, Beijing, 100084 China; 9 Tsinghua-Berkeley Shenzhen Institute (TBSI), Center for Biotechnology and Biomedicine and Shenzhen Key Laboratory of Gene and Antibody Therapy, State Key Laboratory of Chemical Oncogenomics, State Key Laboratory of Health Sciences and Technology, Institute of Biopharmaceutical and Health Engineering (iBHE), Shenzhen International Graduate School, Tsinghua University, Shenzhen, 518055 China; 10 Henry Fok College of Biology and Agriculture, Shaoguan University, Shaoguan, 512005 China

**Keywords:** Orchid, Whole-genome sequences, *Apostasia shenzhenica*, *Dendrobium catenatum*, *Phalaenopsis equestris*, Database

## Abstract

**Background:**

The Orchid family is the largest families of the monocotyledons and an economically important ornamental plant worldwide. Given the pivotal role of this plant to humans, botanical researchers and breeding communities should have access to valuable genomic and transcriptomic information of this plant. Previously, we established OrchidBase, which contains expressed sequence tags (ESTs) from different tissues and developmental stages of *Phalaenopsis* as well as biotic and abiotic stress-treated *Phalaenopsis*. The database includes floral transcriptomic sequences from 10 orchid species across all the five subfamilies of Orchidaceae.

**Description:**

Recently, the whole-genome sequences of *Apostasia shenzhenica*, *Dendrobium catenatum*, and *Phalaenopsis equestris* were de novo assembled and analyzed. These datasets were used to develop OrchidBase 4.0, including genomic and transcriptomic data for these three orchid species. OrchidBase 4.0 offers information for gene annotation, gene expression with fragments per kilobase of transcript per millions mapped reads (FPKM), KEGG pathways and BLAST search. In addition, assembled genome sequences and location of genes and miRNAs could be visualized by the genome browser. The online resources in OrchidBase 4.0 can be accessed by browsing or using BLAST. Users can also download the assembled scaffold sequences and the predicted gene and protein sequences of these three orchid species.

**Conclusions:**

OrchidBase 4.0 is the first database that contain the whole-genome sequences and annotations of multiple orchid species. OrchidBase 4.0 is available at http://orchidbase.itps.ncku.edu.tw/

## Background

The Orchid family is the largest families of the monocotyledons and an economically important ornamental plant worldwide. The orchid is a valuable evolutionary model organism with an unparalleled diversity of innovative vegetative, floral and ecological features. They have colonized successfully almost all habitats on earth. The reasons of the orchid’s dramatic diversification have been associated to the specific interaction between the orchid flower and pollinators [1], rapid and successive interplay between natural selection and drift [2], symbiotic relationship between orchid and fungi [3], crassulacean acid metabolism (CAM) and epiphytic growth [4]. The speciation rate of orchids is suggested to be exceptionally high [5]. New species of orchids keep being discovered worldwide implying that the evolution of orchids is still ongoing.

**Fig. 1:**
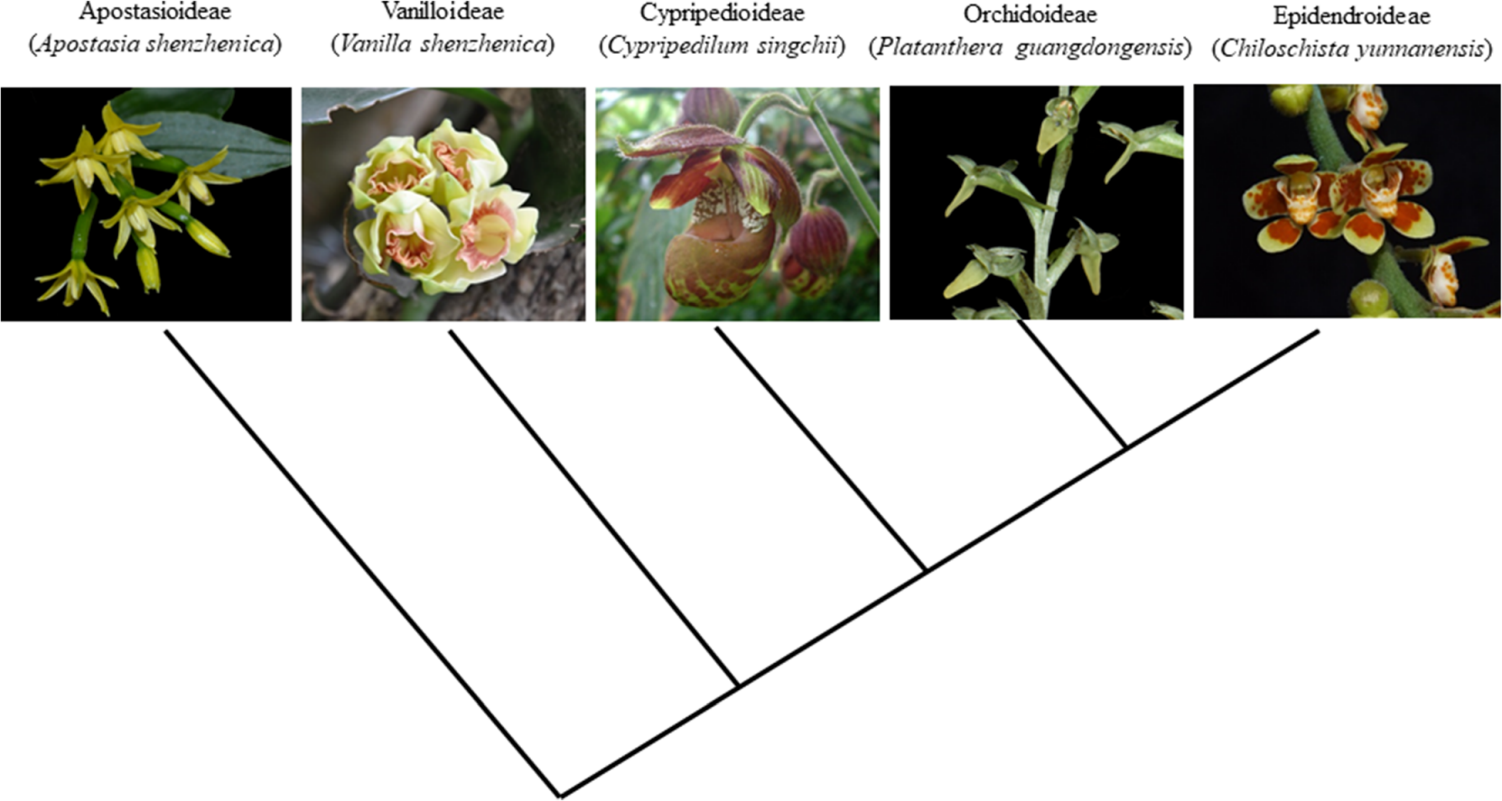
Phylogeny of five subfamilies in Orchidaceae

Containing more than 900 genera and 27,000 species [6], the Orchidaceae belonging to class Liliopsida, order Asparagales, is composed of five subfamilies including Apostasioideae Cypripedioideae, Epidendroideae, Orchidoideae and Vanilloideae (Fig. 1). Orchids have unique reproductive strategies that contribute to their successful radiation. These include pollination-triggered ovary/ovule development, mature pollen grains aggregated as pollinia, micro- and mega-gametogenesis with highly synchronized timing for effective fertilization, and the dispersal of millions of immature embryos from mature pods [7]. Several orchid species have been used as model species for plant science research. In especial, because *Phalaenopsis* and their hybrids are important for the orchid breeding and the availability of horticultural mutants, the *Phalaenopsis* plants are often chosen for the orchid development study [8–11]. Species of *Phalaenopsis* are found throughout the islands of the Pacific Ocean and the tropical Asia. *Phalaenopsis equestris* and *Phalaenopsis aphrodite* subspecies formosana, two native species in Taiwan, are often chosen as parents for breeding commercial cultivars. *P. equestris* has several beneficial traits such as branches with abundant colorful flowers and numerous spikes. *P. equestris* is a diploid plant and the estimated haploid genome size 1.6 Gb, which is relatively small in *Phalaenopsis* [12, 13]. *P. equestris* has 38 chromosomes that are small and uniform in size (< 2 μm). The fundamental studies and genomic sequences availability have laid the basis for *P. equestris* to be the first whole-genome sequenced orchid plant [8]. *Dendrobium* is the third largest genus of Orchidaceae. *Dendrobium* is a fascinating group of orchids because of their diverse floral architectures, fleshy stems, and synthesis of many kinds of polysaccharides [14]. The fleshy stem of *Dendrobium catenatum* contains various kinds of polysaccharides. Many of these polysaccharides have medicinal applications, such as immuno-enhancing, anti-inflammatory, antioxidant and anti-glycation activities [14]. *Apostasia shenzhenica* is a representative of one of two genera, Apostasia and Neuwiedia, that form a sister clade to the rest of the Orchidaceae. Apostasioideae possess several morphologically unique characteristics different from other orchids. The most remarkable one is their floral morphology. Apostasia shows an undifferentiated labellum at the adaxial side of second floral whorl and relatively simple gynostemium at the center of the flower [15].

Previously, we established OrchidBase to accommodate and manage the transcriptome sequences in 11 cDNA libraries, generated from various tissues, including inflorescence and flower buds, vegetative tissue, leaf, developing seed, protocorm, cold-treated and pathogen-inoculated plantlets of the three orchid species (*P. equestris*, *P. bellina* and *P. aphrodite* subsp. Formosana) [16]. The second version of OrchidBase included the floral transcriptomes of 10 orchid species from each of the five Orchidaceae subfamilies: *Neuwiedia malipoensis* and *Apostasia shenzhenica* (Apostasioideae); *Paphiopedilum armeniacum* and *Cypripedium singchii* (Cypripedioideae); *Cymbidium sinense* and *Phalaenopsis equestris* (Epidendroideae); *Hemipilia forrestii* and *Habenaria delavayi* (Orchidoideae); *Vanilla shenzhenica* and *Galeola faberi* (Vanilloideae) [17].

In year 2015, the genome of *P. equestris* was sequenced via a whole-genome shotgun strategy. Its genome size is estimated to be 1.16 Gb, which contains 29,431 predicted protein-coding genes [18]. This species is also the first whole-genome–sequenced water-conserving CAM (crassulacean acid metabolism) plant. CAM means an important elaboration of photosynthetic carbon fixation that allows chloroplast-containing cells to fix CO2 initially at night using phosphoenolpyruvate carboxylase (PEPC) in the cytosol. The genome information of *P. equestris* was used to construct OrchidBase 3.0 [19]. In year 2016, the whole genome of *D. catenatum* was sequenced by Illumina HiSeq 2000 platform [14]. In year 2017, the primitive orchid *A. shenzhenica* was whole genome sequenced by using several different approaches including PacBio, Illumina, and 10X genomics technologies [15]. In the work of Zhang et al. [15], the quality of *P. equestris* and *D. catenatum* assembled genome was respectively improved by adding sequence reads generated by both PacBio and 10X genomics technologies. Owing to OrchidBase 3.0 contained the limited old version information of *Phalaenopsis* genome published in 2015, we update the new version genome of *P. equestris*, and added genomic information of two other orchid species, *D. catenatum* and *A. shenzhenica*, in OrchidBase 4.0. Useful annotation information and easy-to-use web interfaces are provided in OrchidBase 4.0 to access comprehensive sequence data.

## Construction and content

### Implementation and architecture

OrchidBase 4.0 is composed of a SQL server database server, a windows application, and a web interface. For storing and managing collected orchid genome sequence information and the annotation data, the SQL Server 2012 system is adopted. The windows application executes sequence analysis, and the C# programs and Perl scripts are applied to parse orchid genome data and construct the database. Several existing tools were used for improving database coverage, system performance, and the user interface. The web interface is constructed using HTML and the Microsoft. NET (framework 4.62). The OrchidBase 4.0 was developed based on Model-View-Controller (MVC) architecture principles by using the ASP.NET MVC 4 framework [20] and Visual C# programming language. The operation system is the IIS 6.0 on the Microsoft Windows Server 2016 Standard. Genome Browser is visualized under Apache web server on the Ubuntu 16.04. The interactive data visualization web page is based on D3.js and ASP.NET MVC. For building a web-based visualization and presenting data in an interactive and convenient way with maximum compatibility, D3.js, the powerful JavaScript toolkit, was applied to create cross-platform vector graphics. The JBrowse, an AJAX-based browser, is applied to navigate orchid genomes [21, 22].

**Fig. 2:**
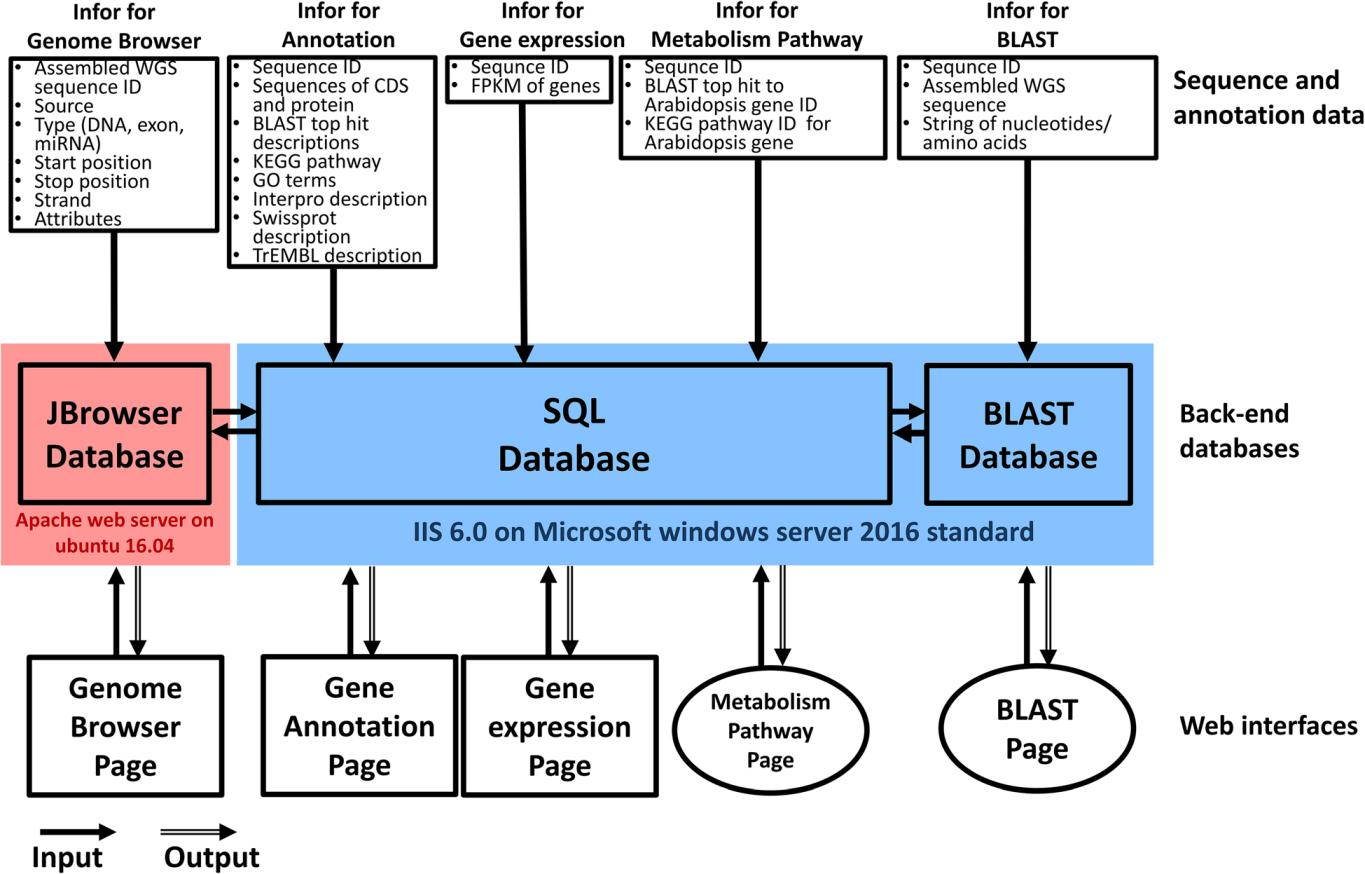
Overview of the database architecture

**Fig. 3:**
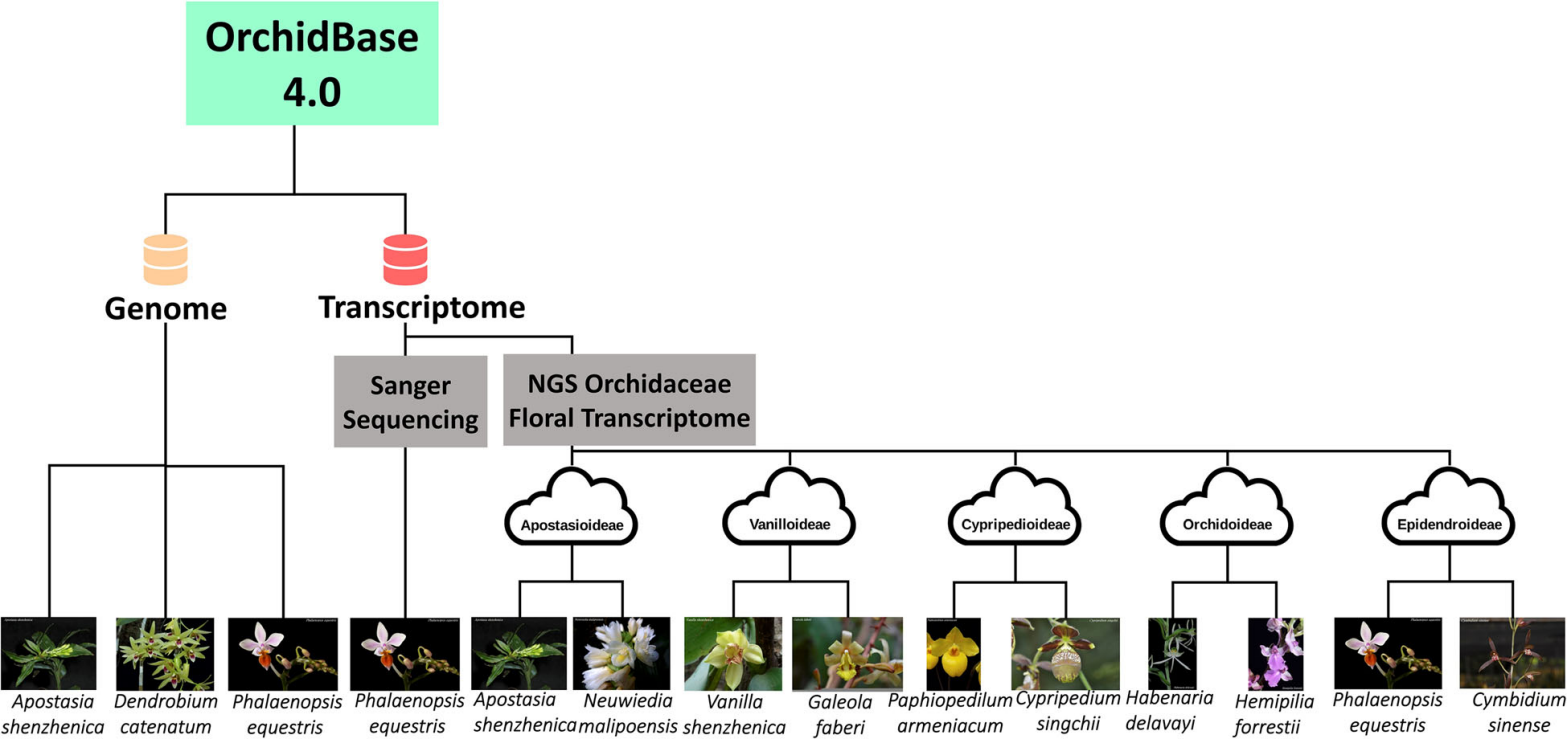
Organizational structure of OrchidBase 4.0 web pages. The OrchidBase 4.0 includes newly added genomic information of three orchid species (Phalaenopsis equestris, Dendrobium catenatum, and Apostasia shenzhenica) and transcriptomic sequences that have been described in the previous version

Figure 2 shows the overview of the database architecture. In addition, the content of the database (data and tools) is summarized in Table 1. The SQL and BLAST database (Fig. 2) are implemented in a virtual machine of a cloud system with one CPU, 2 TB hard disk, and 16 GB RAM. Genome Browser is equipped in the hardware of a workstation with one CPU (48 cores), 2 TB hard disk, and 346 GB RAM (Fig. 2). Figure 3 shows the feature diagram of the OrchidBase 4.0 which including genome (newly created in this version) and transcriptome (described in OrchidBase and OrchidBase 2.0) information. The OrchidBase 4.0 simplifies the workflow for large and complex orchid genome data analysis and visualization. OrchidBase 4.0 is an open-access, web-available portal that integrates the available data for the genomes of the three orchid species and related transcriptomic information.

**Table 1:** Summary of data and tools that could be browsed and used for the three orchid species (*Phalaenopsis equestris*, *Dendrobium catenatum*, and *Apostasia shenzhenica*)

Genome browser	Scaffold ID, Scaffold sequence, Gene model, miRNA
**Gene annotation**	Gene ID, Gene sequence, BLAST top hit descriptions, KEGG pathway, GO terms, Interpro description, Swissprot description, TrEMBL description
**Gene expression**	Gene ID, FPKM value in various tissues
**Metabolism pathway**	Gene ID, Genes mapped to the KEGG pathways
**BLAST tools**	BLASTN, BLASTX, tBLASTX, BLASTP, tBLASTN

### Expanded database content

The raw data and whole genome-assembled scaffold sequences of *Phalaenopsis equestris* (BioProject PRJNA192198 and PRJNA389183) were downloaded from the NCBI database. The Bioproject PRJNA262478 containing raw data and whole genome-assembled scaffold sequences of *Dendrobium catenatum* were also downloaded. Genome sequences and whole-genome assembly of *Apstasia shenzhenica* included in BioProject PRJNA310678 were fetched. Statistics of these three orchid genomes is shown in Table 2.

**Table 2:** Comparison of the assembled genomes among the three orchid species collected in the OrchidBase 4.0

Orchid species	Assembled genome size	N50 length of scaffold (Mb)	N50 length of contig (Kb)	Number of predicted genes	Reference
Phalaenopsis equestris	1.13 Gb	1.22	45.8	29,545	Zhang et al. [15]
Dendrobium catenatum	1.12 Gb	1.06	51.7	29,257	Zhang et al. [15]
Apstasia shenzhenica	349 Mb	3.03	80.1	21,841	Zhang et al. [15]

Based on these datasets, predicted protein-coding genes and translated amino acid sequences were annotated by combining homology-based prediction, de novo gene prediction, and RNA sequence-aided prediction [15]. Each predicted gene is assigned to a specific Gene ID. The specific genes could be selected to investigate their annotated functions of biological processes.

The transcriptomics data were downloaded from BioProjects PRJNA288388, PRJNA304321, and PRJNA348403. For providing expression profile of each orchid gene, all RNA-seq reads were mapped to the predicted genes and counted FPKM values for each gene in the various tissues and different developmental stages. All of this biological information has been integrated into the OrchidBase 4.0.

## Utility and discussion

### Searching the genome information of the three orchid species in the database

**Fig. 4:**
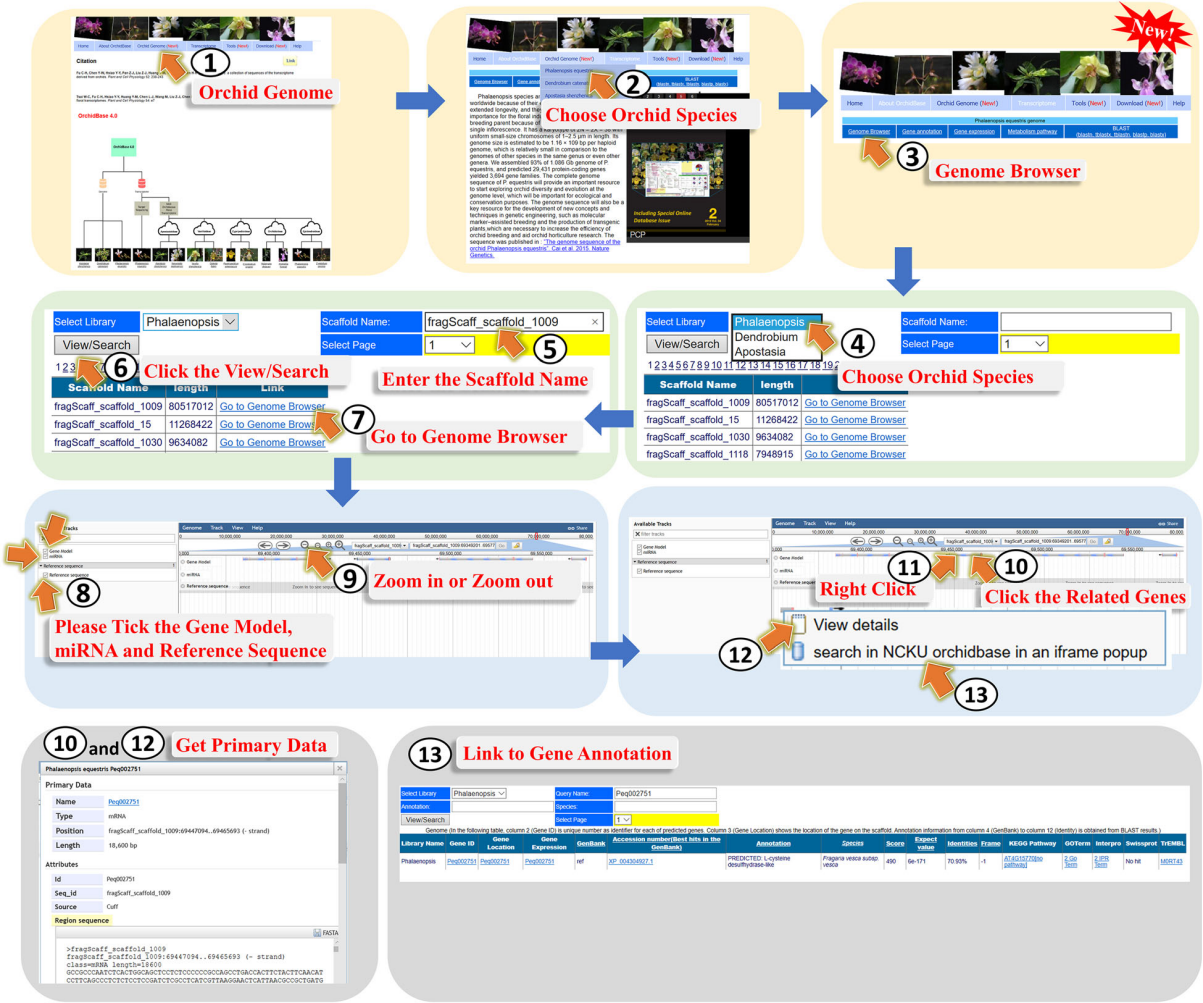
A step-by-step guide for the “Genome Browser” page

The *A. shenzhenica*, *D. catenatum*, and *P. equestris* genome information in OrchidBase 4.0 can be searched to acquire the assembled scaffolds and predicted gene information. Through the web interface, the three orchid genome information contained in OrchidBase 4.0 could be freely accessed. The information can be accessed via the “Orchid Genome” icon (Fig. 4, step 1). With the web interface, a page allows users to select one of the three orchid genomes (Fig. 4, step 2). Users then could access the five webpages (gene annotation, genome browser, metabolism pathway, gene expression, and BLAST) for querying the genome and retrieve the gene information in the selected orchid genome (Fig. 4, step 3).

### Database user protocol

#### Genome Browser page

Genome browser is a graphical interface for displaying information of the genomic data. JBrowse browser, a next-generation genome browser [22], is used in OrchidBase 4.0. The JBrowse tool provides all the assembled scaffolds, which enables the user to access any scaffold region of the chosen orchid genome (Fig. 4, step 4). The webpage lists all the scaffolds for the user to select (Fig. 4, step 5). Click the “View/Search” icon, the selected scaffold‘s information could be shown (Fig. 4, step 6), and then further lead to the webpage to navigate the selected scaffold detailed data (Fig. 4, step 7). The location of genes as well as the intron/exon structure of genes could be visualized (Fig. 4, step 8 to 12). In addition, the miRNA annotation was integrated in the genome sequence (Fig. 4, step 8). Clicking a gene opens a popup with links for downloading the CDS sequences or gene annotation information (Fig. 4, step 10 to 12). Gene model presented in the Genome Browser interface could jump to the “Gene annotation” page. (Fig. 4, step 13).

#### Gene annotation page.

**Fig. 5:**
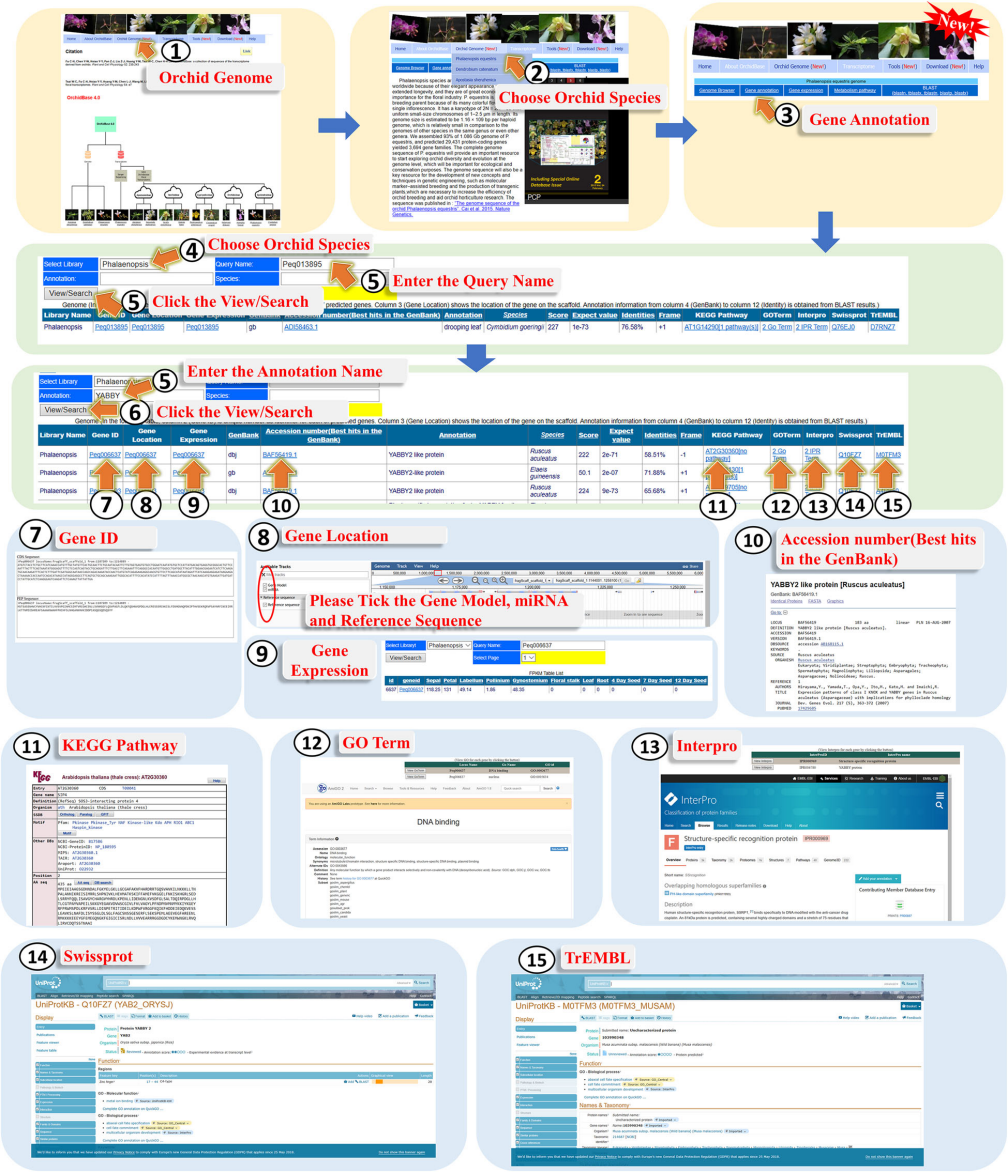
A step-by-step guide for the “Gene annotation” page

The “Gene annotation” page lists the Gene ID, the location of the corresponding scaffold, best hit of the homologs, E-value, KEGG pathway mapping, gene ontology (GO) terms, Interpro, Swissprot, and TrEMBO classification (Fig. 5). Users could access this page through step 1 to step 3 of Fig. 5. Through the web interface, users could query the target information by selecting the orchid species and inputting and/or submitting keywords or a Gene ID to the server (Fig. 5, step 4 to step 6). The gene sequence and the annotated information managed in the relational database are shown in the web interface in response to a query. Users could get the sequence of the selected gene from the Gene ID (Fig. 5, step 7), internally link to “Genome Browser” page from the Gene location (Fig. 5, step 8), get the FPKM value of the gene expression (Fig. 5, step 9), link to GenBank from the Accession number (Fig. 5, step 10), KEGG database from KEGG pathway (Fig. 5, step 11), GO database from GO term (Fig. 5, step 12), Interpro database from Interpro (Fig. 5, step 13), Swiss-Prot database from Swissprot (Fig. 5, step 14), and TrEMBL database from TrEMBL (Fig. 5, step 15).

#### Gene expression page and Metabolism pathway page.

**Fig. 6:**
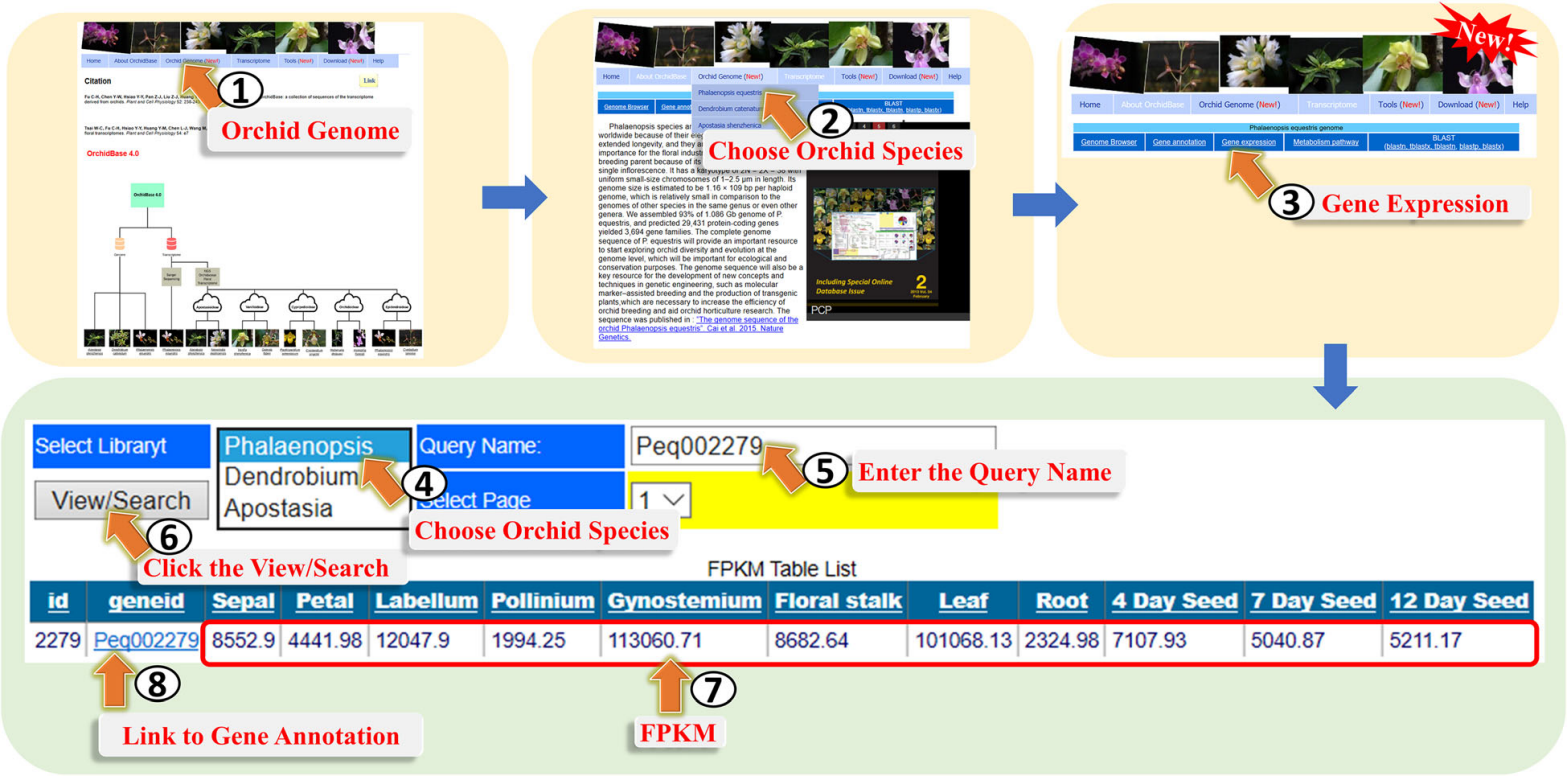
A step-by-step guide for the “Gene expression” page

The list of annotated genes with the FPKM value can be explored in the “Gene expression” page (Fig. 6). Users could reach this page through step 1 to step 3 of Fig. 6. Users can select the species and input a Gene ID to find the expression of the gene with the FPKM value at various tissues and different developmental stages (Fig. 6, step 4 to step 7). The Gene ID in this page internally links to “Gene annotation” page (Fig. 6, step 8).

**Fig. 7:**
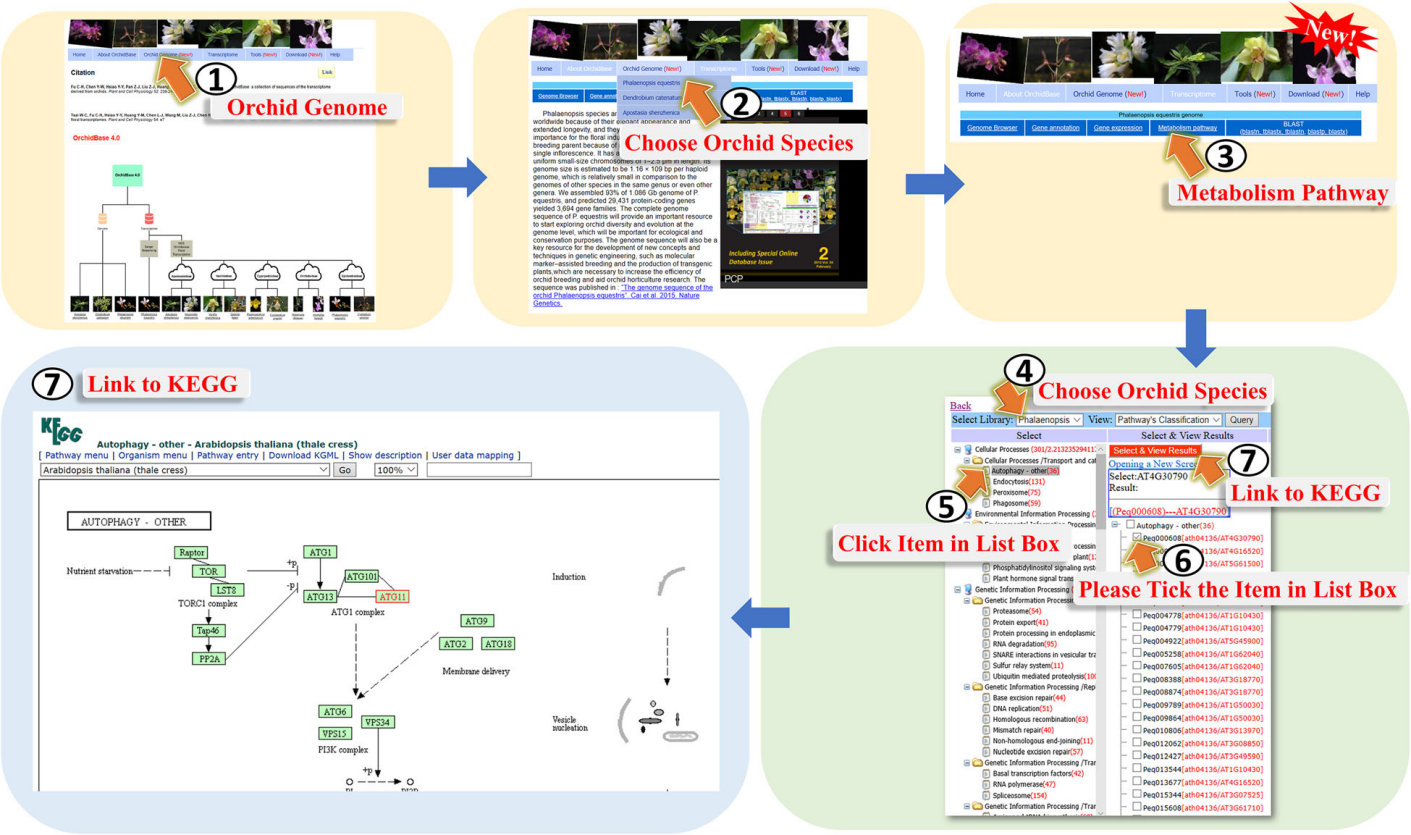
A step-by-step guide for the “Metabolism pathway” page

The “Metabolism pathway” page provides information for the selected genes involved in the KEGG pathways (Fig. 7). Users could go to this page through step 1 to step 3 of Fig. 7. When selecting a species (Fig. 7, step 4) and clicking on a pathway name (Fig. 7, step 5), the panel contents are substituted to portray the Gene IDs involved in the pathway. Users then could select the specific Gene IDs and click the “Select & View Results” icon, and the image displays red-colored enzymes found in the KEGG database (Fig. 7, step 6 to step 7). The colored pathway image is interactive for accessing the KEGG database to explore more information (Fig. 7, step 7).

#### (IV) BLAST page

**Fig. 8:**
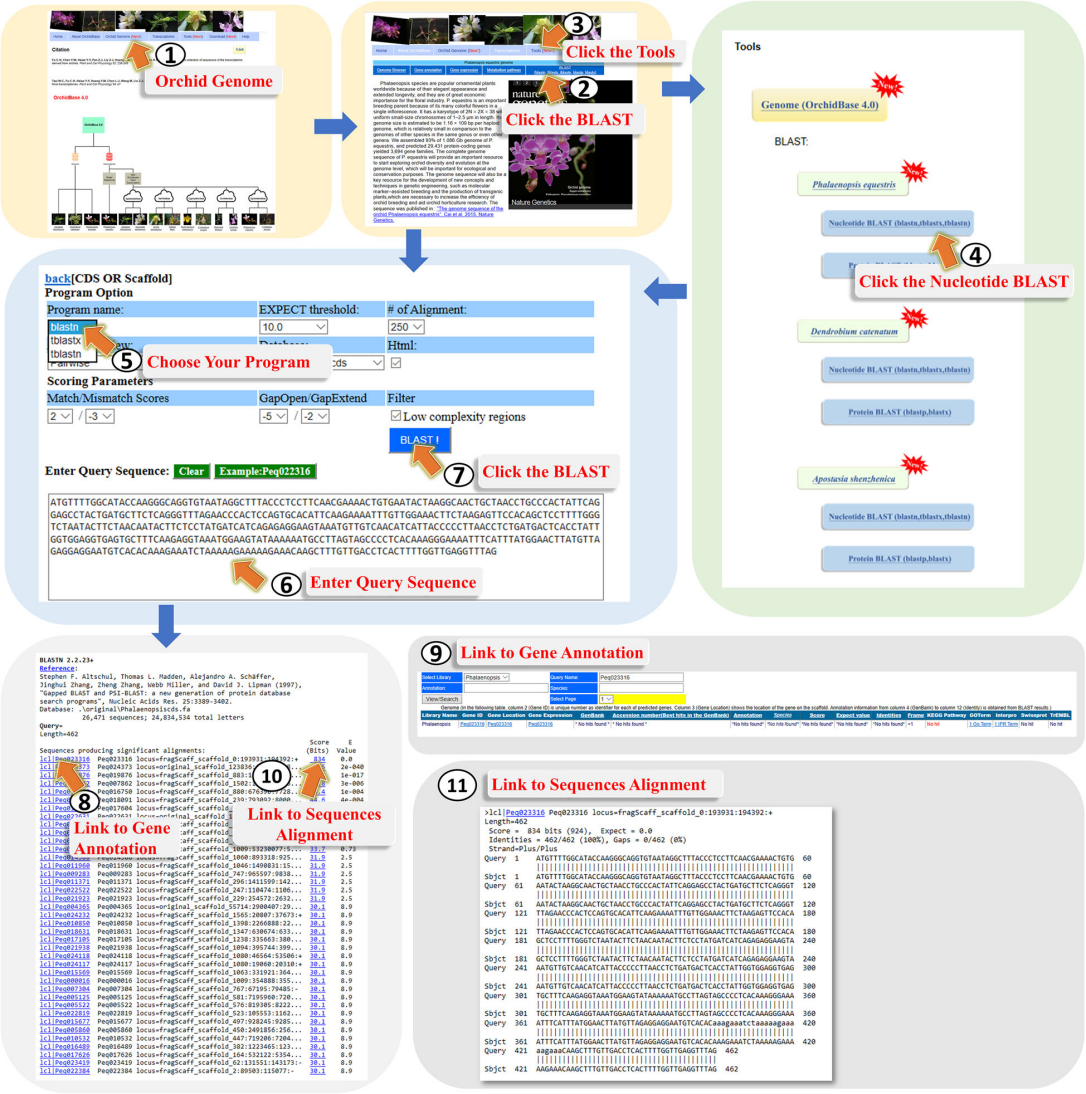
A step-by-step guide for using the “BLAST” tool

To help users perform sequence alignment, OrchidBase 4.0 provides a graphic user interface for users to run BLAST (Fig. 8). The assembled scaffold sequences, predicted gene and protein sequences can be used for BLAST searches [23]. Users could access this function by two ways: through step 1 to step 2 or through step 3 to step 4 of Fig. 8. Users can perform similarity searches of an input sequence against coding DNA sequences, predicted protein sequences, and assembled genome sequences using BLAST search tools (BLASTx, tBLASTx, BLASTp, BLASTn, tBLASTn) (Fig. 8, step 5). The sequences can be submitted by pasting the sequences in the webpage (Fig. 8, step 6 to step 7). Users can set appropriate parameters or simply select the default parameters to run the search. The result of BLAST search contains the lists of gene IDs (Fig. 8, step 8 and step 10), a link to “Gene annotation” page (Fig. 8, step 9) and the details of the alignment results (Fig. 8, step 11).

## Conclusions

The addition and integration of orchid whole genomic sequences with detailed annotation information and easy-to-use web interfaces in OrchidBase 4.0 allow users to efficiently find target genes, such as floral development-related genes [24], floral pigmentation pattern-related genes [25], TCP transcription factor genes [11], and transposable element [26]. In addition, the orchid genome sequence has been supplied valuable information for plant genome evolution and comparative genomic studies [15, 27]. The OrchidBase 4.0 enables using genomic data to understand the fundamental biology of orchids. In addition, with increases in the amount of data from high-throughput technologies for genetic and physical map construction, both types of maps will be available for orchids in the near future. At present, several whole-genome sequencing projects for species in different subfamilies of Orchidaceae are ongoing. OrchidBase 4.0 will be updated continuously and more -omic information and analysis tools will be included for comprehensive analysis of the orchid gene function and genome evolution.

### Availability and requirements

Project name: OrchidBase 4.0.

Project home page: http://orchidbase.itps.ncku.edu.tw/

Operating system(s): Microsoft Windows Server 2016 Standard.

Programming language: ASP.NET and Visual C#.

Other requirements: none required.

License: none required.

Any restrictions to use by non-academics: no restriction.

## Data Availability

The raw data and whole genome-assembled scaffold sequences for *Phalaenopsis equestris* (BioProjects PRJNA192198 and PRJNA389183), *Dendrobium catenatum* (BioProject PRJNA262478), and *Apostasia shenzhenica* (BioProject PRJNA310678) were downloaded from the National Center for Biotechnology Information (NCBI) database. The transcriptomics data were downloaded from BioProjects PRJNA288388, PRJNA304321, and PRJNA348403.

## Abbreviations

A. shenzhenica: Apstasia shenzhenica
CAM: Crassulacean Acid Metabolism
D. catenatum: Dendrobium catenatum
ESTs: Expressed Sequence Tags
FPKM: Fragments Per Kilobase of transcript per Millions mapped reads
KEGG: Kyoto Encyclopedia of Genes and Genomes
GO: Gene Ontology
*P. equestris*: Phalaenopsis equestris
SQL: Structured Query Language
